# CD4^+^CD25^+^ Cells Are Essential for Maintaining Immune Tolerance in Chickens Inoculated with Bovine Serum Albumin at the Late Stage of Embryonic Development

**DOI:** 10.3390/vetsci7040150

**Published:** 2020-10-03

**Authors:** Xiaoxue Yu, Yufang Meng, Pengyu Pan, Liuan Li, Lei Rui, Zandong Li

**Affiliations:** 1 Tianjin Key Laboratory of Agricultural Animal Breeding and Healthy Husbandry, College of Animal Science and Veterinary Medicine, Tianjin Agricultural University, Tianjin 300384, China; mengyihui7799@163.com (Y.M.); ppypp530@163.com (P.P.); anliuli2003@163.com (L.L.); 2 State Key Laboratories for Agrobiotechnology, Department of Biochemistry and Molecular Biology, College of Biological Science, China Agricultural University, Beijing 100193, China; ruilei@cau.edu.cn

**Keywords:** Gallus gallus, CD4^+^CD25^+^ cells, immune tolerance

## Abstract

In this study, the role of chicken CD4^+^CD25^+^ cells during induced immunotolerance was tested. Properties of chicken CD4^+^CD25^+^ cells sorted by flow cytometry were analyzed. Results showed that chicken CD4^+^CD25^+^ cells express IL-10, TGF-β highly and suppress proliferation of CD4^+^CD25^−^ cells in vitro. To induce immunotolerance, embryos were inoculated with bovine serum albumin (BSA) via an intravascular route on embryo incubation day 20 (EID20), and after hatching chicks experienced BSA immunization four times at 7-day intervals. Serum anti-BSA antibodies and CD4^+^CD25^+^ cell ratio was analyzed. Results showed that humoral tolerance was obtained and the CD4^+^CD25^+^ cell percentage in peripheral blood lymphocytes increased along with this progress. Injection of anti-chicken CD25 antibody via an intravascular route on EID16 is applied to block CD4^+^CD25^+^ cells, and the CD4^+^CD25^+^ cell ratio decreased significantly up to 35 d post-hatch. Based on the above, injections of anti-chicken CD25 antibody on EID16 and BSA on EID20 were carried out sequentially, and tolerance level was contrasted to the BSA-injection group. Data revealed the anti-BSA antibodies increased significantly in the CD4^+^CD25^+^ cell-blocked groups indicating that immune tolerance level was weakened. In conclusion, chicken CD4^+^CD25^+^ cells are essential in maintaining induced immune tolerance.

## 1. Introduction

The poultry bioreactor which can produce foreign proteins based on poultry transgenic technology has great potential economic benefits [1,2]. In order to prevent the immune system in avians from recognizing and removing foreign proteins, it is particularly important to induce the immune tolerance and study the mechanisms of tolerance of birds. Moreover, chickens are suited for immune tolerance studies because their ontogenesis is different from mammals in that avian embryos develop outside the mother.

There have been studies on the methods and mechanisms of immune tolerance induction artificially in chickens. Studies of Tempelis et al. [3] and Wolfe et al. [4] showed that intraperitoneal or intravenous injections of about 11 mg of bovine serum albumin into newly hatched chicks can induce tolerance. Tolerance was also observed after injection of human serum albumin (HSA) into chicken embryo yolk sacs [5] or after hatched [6]. It is not difficult to find that a fairly huge amount of protein antigen is usually needed to be injected into hatched chicks initially in order to produce immunological unresponsiveness. Ontogenesis in avian species is different from that in mammals because avian embryos develop outside the mother and this provides advantageous conditions for inoculation with antigens during embryogenesis. Previous studies in our lab have shown that microinjection of BSA via the chicken embryonic blood vessel can induce immune tolerance [7,8]. However, which kind of immune cell subgroup played roles is not explicit.

The immunological tolerance is based on the B and T lymphocytes being unresponsive to antigens which they encountered during the maturation process, which is as known as the clonal selection theory of acquired immunity [9]. Regulatory T cells (Tregs) play an indispensable role in maintaining immunological tolerance [10]. CD4^+^CD25^+^ cells in chickens have regulatory-T-cell-like properties similarly to those in mammals, producing high amounts of pro-inflammatory cytokines and suppressing the proliferation of naïve T cells in vitro [11]. Previous experimental data of our laboratory had shown that the percentage of CD4^+^ T cells among CD3^+^ T cells increased significantly in the tolerance group induced by BSA injection. In this study, we further examined if CD4^+^CD25^+^ cells were involved in this process.

Blocking a specific cell subset by antibodies is widely used for exploring the function of them. Results from Onizuka et al. [12] and Wen et al. [13] showed that administration of anti-CD25 monoclonal antibody (mAb) can reduce the number of CD4^+^CD25^+^ cells in mice. Debora Vignali et al. [14] observed an almost complete depletion of Treg cells after injection of anti-CD25 mAb in patients with type 1 diabetes who received pancreatic islet transplantation. In chickens, Shanmugasundaram et al. [15] found that CD4^+^CD25^+^ cells started to appear at EID15 in thymus. Furthermore, the studies of them have demonstrated that injection of anti-CD25 mAb through the amniotic route could block the CD4^+^CD25^+^ cells in chickens [16,17]. However, this requires a relatively large number of antibodies. In this study, a more direct and effective approach was carried out to block chicken CD4^+^CD25^+^ cells by micro-injecting anti-chicken CD25 mAb via embryonic vasculature.

In summary, to reveal the role of chicken CD4^+^CD25^+^ cells in the induced immune tolerance, we injected anti-CD25 mAb and BSA successively during embryonic development and analyzed humoral tolerance levels after hatching.

## 2. Materials and Methods

### 2.1. Ethics Statements

The animal welfare and experimental procedures adhered to the Institutional Guidelines of the Care and Use of Laboratory Animals at Tianjin Agricultural University (approved code: TJAU2018009, 7 April 2018). All efforts were made to minimize suffering.

### 2.2. Fertilized Eggs and Chickens

White leghorn (Gallus gallus domesticus) strain fertilized eggs were purchased from the Experimental Station of the China Agricultural University and incubated at 37.6 °C in a forced air incubator at 53–63% relative humidity with 90° tilting once per hour (P-008B Biotype, Showa Furanki, Saitama, Japan). After hatching, chicks were housed in isolators and water and food were freely available.

### 2.3. Proteins and Antibodies

BSA was purchased from Sigma–Aldrich (St. Louis, MO, USA). Goat anti-chicken IgY and IgM peroxidase-conjugated antibodies were purchased from Southern Biotechnology Associates (Birmingham, AL, USA). Goat anti-chicken IgA peroxidase-conjugated antibody was purchased from Bethyl Laboratories, Inc. (Montgomery, TX, USA).

The fluorescein isothiocyanate (FITC)-conjugated mouse anti-chicken CD4 antibody was purchased from SouthernBiotech (Birmingham, AL, USA), and the isotype is mouse IgG1k. The mouse anti-chicken CD25 mAb and RPE-conjugated anti-chicken CD25 mAb was produced as previously reported [18].

### 2.4. Quantitative Real-Time PCR Analysis of Gene Expression

The quantitative real-time PCR was performed in 15 μL of LightCycler^@^ 480 SYBR Green I Master Mix (Roche, Rotkreuz, Switzerland) with the LightCycler^@^ 480 Real-Time PCR System (Roche). PCR cycles were set at the following conditions: 10 min at 95 °C; 40 cycles of 95 °C for 15 s and 55 °C for 1 min; 95 °C for 15 s and 60 °C for 15 s and 95 °C for 15 s to get a final melting curve; cooling at 40 °C for 30 s. Fluorescent emission was detected during the extension step. The expressions of genes are calculated as relative expression with *gapdh* which is the housekeeping gene. The primers used are shown in Table 1.

### 2.5. Suppression Assay

A suppression of T cell proliferation assay was employed to assess the suppressive properties of thymic CD4^+^CD25^+^ cells. The suppression of T cell proliferation assay is a coculture assay between Tregs and carboxyfluorescein succinimidyl amino ester (CFSE)-labeled naïve T cells. Coculture assay was performed by co-incubating 3 × 10^4^ CFSE-labeled CD4^+^CD25^−^ cells with CD4^+^CD25^+^ cells at a ratio of either 1:1 or 1:0 in three replications (n = 3). The CFSE-labeled CD4^+^CD25^−^ cells were measured at 66 h of coculture after gating on CFSE-stained cells. The non-proliferated cell percentage in the coculture group was determined by flow cytometry after gating on the CFSE-positive responder cells.

### 2.6. Microinjection via Embryonic Blood Vessel and Post-Hatch Immunization

BSA injection: A small window was opened in the air chamber and 0.75 mg of BSA was directly injected into the embryonic blood vessels on EID20 (Supplementary Materials).

Anti-chicken CD25 antibodies injection: A small window was opened in the air chamber and approximately 15 µg of anti-chicken CD25 antibodies was injected into the embryonic blood vessels on EID16 (Supplementary Materials).

The microinjection is conducted as follows: Prepare the fertilized eggs. The fertilized eggs were incubated to EID16 or EID20. Make a small window on the blunt end of the egg using a dental drill gently and steadily. Drop a little PBS on the eggshell membrane. Find the blood vessel, and inject the substance with micro-glass capillary needle under the microscope. Seal the small window with a sealing film. Put the egg back in the incubator to continue to hatch.

We divided the eggs into four groups: group 1 (BSA injected on EID20), group 2 (anti-chicken CD25 antibodies injected on EID16 and BSA injected on EID20), group 3 (anti-chicken CD25 antibodies injected on EID16), group 4 (PBS injected on EID16). The window was sealed, and the eggs were incubated with the blunt end facing upwards until hatching. After hatching, the chickens were housed in the same conditions; water and food were freely available. Chickens of group 1 and group 2 were immunized four times with 200 µg BSA per chicken. The initial immunization was carried out by dorsal hypodermic injection with BSA emulsified with an equal volume of Freund’s Complete Adjuvant (Sigma, St. Louis, MO, USA) at 7-days post-hatch. Then, three injections of BSA emulsified with an equal volume of Freund’s Incomplete Adjuvant (Sigma, St. Louis, MO, USA) were administered at 7-day intervals. Peripheral blood lymphocytes and serum samples were isolated before immunization.

### 2.7. Serum Sample Collection and Enzyme-Linked Immunosorbent Assay

The levels of specific anti-BSA antibodies in the sera were determined by ELISA according to previous methods. Peripheral blood was obtained by venipuncture, and serum was separated by coagulation at 37 °C for 1 h, followed by centrifugation (10,000× *g*, 10 min). Equal amount of blood from 3–5 birds were pooled to obtain one sample, and three or four replications (n = 3 or 4) were made to be analyzed. ELISA plates (96-wells; Sigma–Aldrich) were coated with 4 µg/mL BSA dissolved in a carbonate-bicarbonate buffer (pH 9.6) and incubated at 4 °C overnight.

The plates were then washed with phosphate buffer (pH 7.4) containing 0.05% Tween-20 (PBST), and the remaining binding sites were blocked with 1% gelatin dissolved in carbonate-bicarbonate buffer (pH 9.6) for 1 h at 37 °C. The plates were washed again with PBST. The serum was diluted in 0.1% gelatin/PBST (pH 7.4) and incubated at 37 °C for 1.5 h. The serum was diluted 1000-fold for the anti-BSA IgY, 300-fold for anti-BSA IgM and 5-fold for anti-BSA IgA antibodies detection. Plates were washed once again and incubated with goat anti-chicken IgY, IgM and IgA peroxidase-conjugated antibodies at 37 °C for 1 h before color development with 3,3′,5,5′-tetramethylbenzidine (Nanjing Jiancheng Bioengineering Institute, Nanjing, China). The levels of anti-BSA antibodies in the serum were calculated from the absorbance at 465 nm as determined by a microplate reader (Tecan, Infinite 200, Salzburg, Austria).

### 2.8. Lymphocytes Preparation and Fluorescence-Activated Cell Sorting (FACS) Analysis

Venous blood lymphocytes from chickens of four groups were collected at 7, 14, 21, 28, and 35 days post-hatch. Peripheral blood mixed with heparin sodium was obtained via venipuncture, and equal amount of blood from 3–5 birds were pooled to obtain one sample, and then lymphocytes were separated in lymphocyte separation medium (TBD, Tianjin, China). Three or four replications (n = 3 or 4) were carried out to be analyzed.

The lymphocytes were stained with RPE-conjugated anti-chicken CD25 antibody and FITC-conjugated anti-chicken CD4 antibody (Southern Biotechnology Associates). A FACS calibur (Becton Dickinson, Franklin, NJ, USA) flow cytometry system was used to conduct flow cytometry and FlowJo (version 7.6.1) software and Microsoft Excel 2010 were used to analyze the data.

### 2.9. Statistical Analysis

Differences in all experiments were analyzed using the Statistical Package for the Social Sciences (SPSS) software (v16.0; SPSS Inc., Chicago, IL, USA) and an independent sample *t*-test. A *p*-value ≤0.05 was considered statistically significant.

## 3. Results

### 3.1. Chicken CD4^+^CD25^+^ Cells Have Suppressive Properties Similar to Those in Mammals

We sorted chicken CD4^+^CD25^+^ cells and CD4^+^CD25^−^ cells by flow cytometry to assess the properties of chicken CD4^+^CD25^+^ cells. Total RNA was extracted from two subpopulations of cells, and then went through reverse transcription, quantitative real-time PCR analysis of *IL-2*, *IL-10*, *TGF-β*. As Table 2 showed, the mRNA of *IL-2* is not detectable in CD4^+^CD25^+^ cells; the mRNA level of IL-10 in CD4^+^CD25^+^ cells is more than 40-fold that in CD4^+^CD25^−^ cells; the mRNA level of *TGF-β* in CD4^+^CD25^+^ cells is more than 10-fold that in CD4^+^CD25^−^ cells. The results indicate that chicken CD4^+^CD25^+^ cells have no *IL-2* expression and high expression of *IL-10* and *TGF-β*.

**Figure 1:**
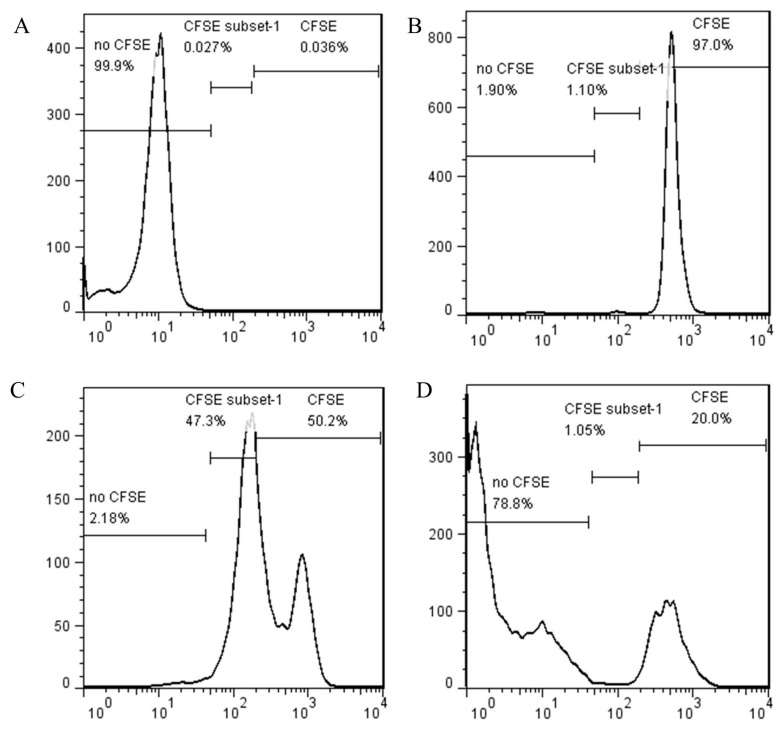
CD4^+^CD25^+^ cells suppress the proliferation of naive T cells: (**A**) negative control: CD4^+^CD25^−^ cells with no CFSE staining; (**B**) positive control: CD4^+^CD25^−^ cells with CFSE staining; (**C**) ConA-stimulated CD4^+^CD25^−^ cells; (**D**) coculture of ConA-stimulated CD4^+^CD25^−^ cells with CD4^+^CD25^+^ cells at a ratio of 1:1. CFSE dilution of CFSE-labeled CD4^+^CD25^−^ cells was measured at 66 h of coculture after gating on CFSE-stained cells. Three gates mean: no CFSE, the percentage of CFSE negative cells; CFSE, the percentage of no-proliferative CFSE positive cells; CFSE subset-1, the percentage of proliferative CFSE positive cell. n = 3, results are representative of two independent experiments.

The mean (± SEM) total proliferated cell percentage in the CD4^+^CD25^−^ cells group with no CD4^+^CD25^+^ cells was 48.5% (Figure 1C). However, the proliferation ratio of CD4^+^CD25^−^ cells decreased remarkably into 6 ± 1% when co-culture with CD4^+^CD25^+^ cells (Figure 1D). The results suggest that chicken CD4^+^CD25^+^ cells have the ability to suppress proliferation of CD4^+^CD25^−^ cells.

### 3.2. Microinjection of BSA on EID20 Can Induce Humoral Immune Tolerance

Chicken embryos were incubated with 0.75 mg of BSA via intravascular injection on EID20. Meanwhile, PBS was injected as the control group. After hatching, four rounds of BSA immunization were carried out and serum antibodies were collected and detected at 14, 21, 28, and 35 days of age.

**Figure 2:**
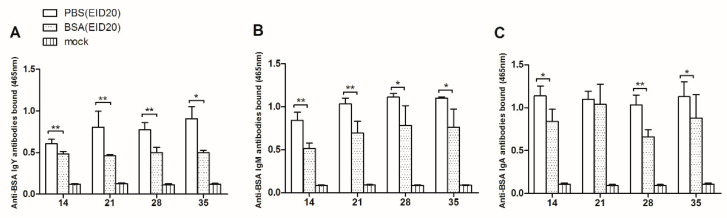
Serum anti-bovine serum albumin (BSA) antibody analysis after hatching: (**A**) the bound anti-BSA IgY antibodies; (**B**) the bound anti-BSA IgM antibodies; (**C**) the bound anti-BSA IgA antibodies. x-axis means chick age of days. y-axis means the OD value at 465 nm. The error bars indicate the SEM. * *p* ≤ 0.05; ** *p* ≤ 0.01.

As showed in Figure 2A, at age of 14 days, the anti-BSA IgY antibodies in the BSA-injection group are significantly less than that in the control group (0.484 ± 0.031, n = 9 versus 0.604 ± 0.056, n = 3). At age of 21 days, the anti-BSA IgY antibodies in the BSA-injection group are significantly less than in the control group (0.463 ± 0.016, n = 9 versus 0.804 ± 0.194, n = 3). At age of 28 days, the anti-BSA IgY antibodies in the BSA-injection group are significantly less than in the control group (0.502 ± 0.061, n = 6 versus 0.772 ± 0.086, n = 3). At age of 35 days, the anti-BSA IgY antibodies in the BSA-injection group are significantly less than in the control group (0.500 ± 0.026, n = 6 versus 0.907 ± 0.145, n = 3).

As showed in Figure 2B, at the age of 14 days, the anti-BSA IgM antibodies in the BSA-injection group are significantly less than in the control group (0.514 ± 0.061, n = 5 versus 0.841 ± 0.096, n = 3). At the age of 21 days, the anti-BSA IgM antibodies in the BSA-injection group are significantly less than in the control group (0.694 ± 0.136, n = 5 versus 1.032 ± 0.067, n = 3). At the age of 28 days, the anti-BSA IgM antibodies in the BSA-injection group are significantly less than in the control group (0.782 ± 0.230, n = 5 versus 1.110 ± 0.044, n = 3). At the age of 35 days, the anti-BSA IgM antibodies in the BSA-injection group are significantly less than in the control group (0.761 ± 0.210, n = 5 versus 1.099 ± 0.015, n = 3).

In Figure 2C, at the age of 14 days, the anti-BSA IgA antibodies bound significantly less in the BSA-injection group than in the control group (0.838 ± 0.143, n = 4 versus 1.136 ± 0.116, n = 3). At the age of 21 days, there was no significant difference between the anti-BSA IgA antibodies in the BSA-injection group and in the control group (1.040 ± 0.233, n = 6 versus 1.099 ± 0.092, n = 3). At age of 28 days, the anti-BSA IgA antibodies bound significantly less in BSA-injection group than in the control group (0.660 ± 0.084, n = 6 versus 1.030 ± 0.116, n = 3). At age of 35 days, the anti-BSA IgA antibodies bound significantly less in BSA-injection group than in the control group (0.878 ± 0.274, n = 6 versus 1.127 ± 0.176, n = 3).

In conclusion, the levels of anti-BSA IgY, IgM, IgA antibodies in BSA-injection group are lower than those in control group 35 days after hatching. These results indicated that injection of BSA at EID20 induced the humoral immune tolerance.

### 3.3. CD4^+^CD25^+^ Cells Increased in the Blood Increased as Induction Progressed

**Figure 3:**
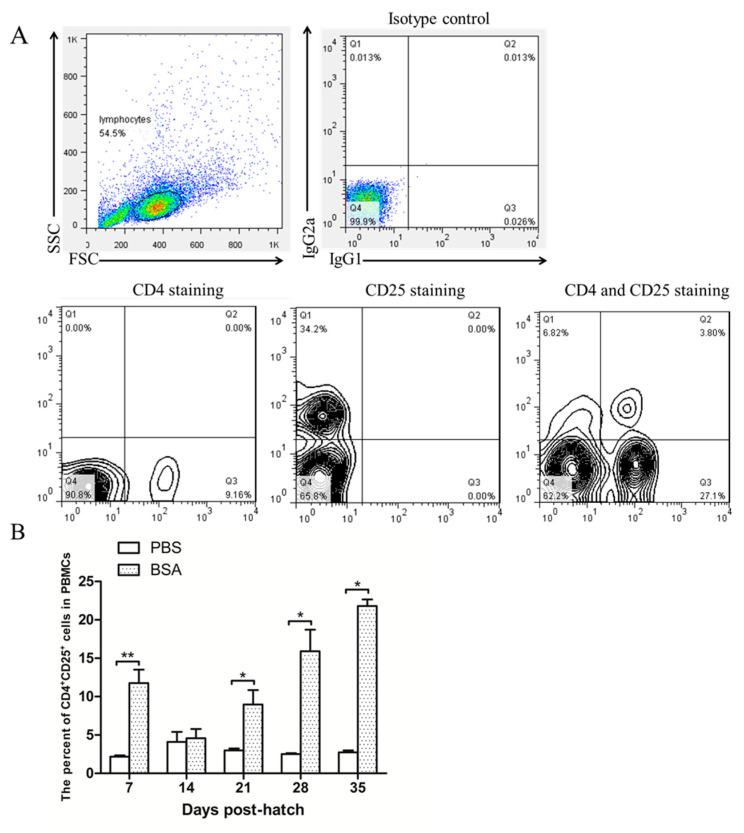
The percentage of CD4^+^CD25^+^ cells by flow cytometry: (**A**) The lymphocytes were grouped and CD4^+^CD25^+^ cells were gated as shown in Figure 3A (up left: lymphocytes group; up right: isotype control; down left: CD25 staining; down middle CD25 staining; down right: CD4 and CD25 staining). (**B**) The percentage of CD4^+^CD25^+^ cells. The error bars indicate the SEM. * *p* ≤ 0.05; ** *p* ≤ 0.01.

Immune tolerance was induced via BSA injection at EID20 as described above. After hatching, blood lymphocytes were isolated for CD4 and CD25 staining at 7-day internals. As shown in Figure 3A, the lymphocytes were grouped depending on forward scatter (FSC) and side scatter (SSC), and CD4^+^CD25^+^ cells were gated by referencing the isotype control, CD4 staining and CD25 staining. CD4^+^CD25^+^ cells were gated as shown in Figure 3B. Figure 3B right showed that the CD4^+^CD25^+^ cells in the peripheral blood lymphocytes increased as this induction progressed. This result indicated that chicken CD4^+^CD25^+^ cells are involved in the induction of immunological tolerance to BSA.

### 3.4. Injection of Anti-Chicken CD25 Antibody on EID16 via Blood Vessel Significantly Blocked CD4^+^CD25^+^ Cells until 35 d Post-Hatch

**Figure 4:**
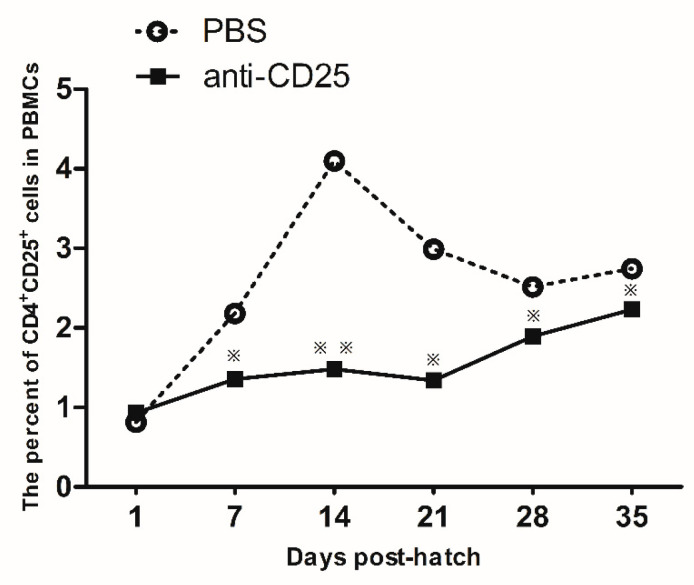
The percentages of CD4^+^CD25^+^ cells in the peripheral blood post-hatch. The error bars indicate the SEM. * *p* ≤ 0.05, ** *p* ≤ 0.01.

Microinjection of the anti-chicken CD25 antibodies was conducted at EID16, and the ratio of CD4^+^CD25^+^ cells was detected by flow cytometry. As shown in Figure 4, the CD4^+^CD25^+^ cells decreased significantly from 7 days post-hatch in the anti-chicken CD25 antibodies injection group compared to the control group. This result showed that microinjection of anti-chicken CD25 antibodies via the blood vessel route was an efficient way to reduce the CD4^+^CD25^+^ cells.

### 3.5. The Humoral Immune Tolerance Was Weakened When CD4^+^CD25^+^ Cells Were Blocked

To explore whether CD4^+^CD25^+^ cells are essential in induction of immune tolerance, we blocked CD4^+^CD25^+^ cells on EID16 prior to BSA injection on EID20. After hatching, chickens recieved four rounds of BSA immunization and serum samples were collected at 7, 14, 21, 28, 35 days post-hatch to detect the level of immune tolerance.

**Figure 5:**
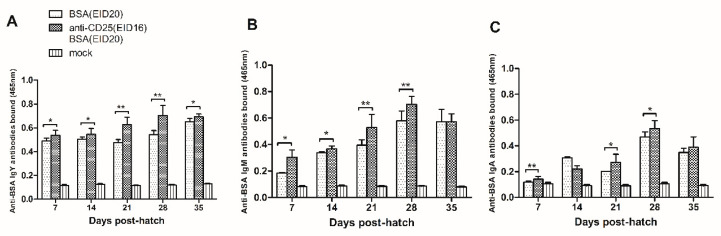
Serum anti-BSA antibody analysis by ELISA: (**A**) the bound anti-BSA IgY antibodies; (**B**) the bound anti-BSA IgM antibodies; (**C**) the bound anti-BSA IgA antibodies. y-axis means the OD value at 465 nm. The error bars indicate the SEM. * *p* ≤ 0.05; ** *p* ≤ 0.01.

As shown in Figure 5A, at 7 days of age, the anti-BSA IgY antibodies in the BSA and anti-chicken CD25 antibodies injection group are significantly more than in the BSA injection control group (0.537 ± 0.043, n = 8 versus 0.488 ± 0.026, n = 5). At 14 days of age, the anti-BSA IgY antibodies in the BSA and anti-chicken CD25 antibodies injection group are significantly more than in the BSA injection control group (0.545 ± 0.051, n = 10 versus 0.505 ± 0.019, n = 6). At 21 days of age, the anti-BSA IgY antibodies in the BSA and anti-chicken CD25 antibodies injection group are significantly more than in the BSA injection group (0.628 ± 0.061, n = 10 versus 0.476 ± 0.026, n = 3). At 28 days of age, the anti-BSA IgY antibodies in the BSA and anti-chicken CD25 antibodies injection group are significantly more than in the BSA injection group (0.702 ± 0.084, n = 11 versus 0.544 ± 0.033, n = 3). At 35 days of age, the anti-BSA IgY antibodies in the BSA and anti-chicken CD25 antibodies injection group are significantly more than in the BSA injection group (0.693 ± 0.023, n = 6 versus 0.652 ± 0.025, n = 3).

As showed in Figure 5B, at 7 days of age, the anti-BSA IgM antibodies in the BSA and anti-chicken CD25 antibodies injection group are significantly more than in the BSA injection group (0.305 ± 0.054, n = 8 versus 0.185 ± 0.004, n = 3). At 14 days of age, the anti-BSA IgM antibodies in the BSA and anti-chicken CD25 antibodies injection group are significantly more than in the BSA injection group (0.366 ± 0.021, n = 7 versus 0.337 ± 0.011, n = 3). At 21 days of age, the anti-BSA IgM antibodies in the BSA and anti-chicken CD25 antibodies injection group are significantly more than in the BSA injection group (0.528 ± 0.100, n = 12 versus 0.395 ± 0.041, n = 6). At 28 days of age, the anti-BSA IgM antibodies in the BSA and anti-chicken CD25 antibodies injection group are significantly more than in the BSA injection group (0.703 ± 0.059, n = 12 versus 0.580 ± 0.074, n = 6). At 35 days of age, the anti-BSA IgM antibodies are equivalent in the BSA and anti-chicken CD25 antibodies injection group and in the BSA injection group (0.571 ± 0.060, n = 12 versus 0.572 ± 0.092, n = 6).

In Figure 5C, in serum collected at 7 days of age, the anti-BSA IgA antibodies in the BSA and anti-chicken CD25 antibodies injection group are significantly more than in the BSA injection group (0.143 ± 0.021, n = 12 versus 0.118 ± 0.008, n = 6). At 14 days of age, there is no significant difference between the anti-BSA IgA antibodies bound in the BSA and anti-chicken CD25 antibodies injection group and in the BSA injection group (0.222 ± 0.024, n = 12 versus 0.306 ± 0.012, n = 6). At 21 days of age, the anti-BSA IgA antibodies bound in the BSA and anti-chicken CD25 antibodies injection group are significantly more than in the BSA injection group (0.271 ± 0.064, n = 9 versus 0.202 ± 0.002, n = 3). At 28 days of age, the anti-BSA IgA antibodies in the BSA and anti-chicken CD25 antibodies injection group are significantly more than in the BSA injection group (0.535 ± 0.061, n = 9 versus 0.469 ± 0.040, n = 6). At 35 days of age, the anti-BSA IgA antibodies bound are equivalent in the BSA and anti-chicken CD25 antibodies injection group and in the BSA injection group (0.389 ± 0.082, n = 12 versus 0.349 ± 0.033, n = 6).

In conclusion, the levels of anti-BSA IgY, IgM, IgA antibodies in anti-CD25 antibodies-injection prior to BSA-injection group are significantly increased than those in BSA-injection control group. These results indicated that injection of anti-chicken CD25 antibodies prior to BSA injection could weaken the level of the humoral immune tolerance to BSA.

## 4. Discussion

Immunological tolerance involves the continuous active controlling and modifying of unnecessarily initiated responses [19]. The immune system will be tolerant to the antigen after maturity if it has been exposed to the antigen during development. Therefore, exposing the embryo to exogenous proteins during the embryo stage is easier to induce tolerance. In previous studies in our lab, we have done extensive microinjection work during chicken embryonic development [8,20,21], and found that injection of BSA via the intravascular route at EID20 induced the immunological tolerance of BSA [8]. BSA injected at EID20 was delivered by blood circulation and distributed in the bursal cortex, spleen and thymus. As is well-known, there is a division of labor and collaboration between T lymphocytes and B lymphocytes [22]. T lymphocytes mediate cellular immunity against bacteria and virus-infected cells. B lymphocytes mediate humoral immunity against circulating pathogens like viruses and foreign proteins. So we determined the amount of anti-BSA antibodies by ELISA to indicate the level of immune tolerance. Previous results in our lab showed that CD4^+^ cells percent increase along the artificially induced immune tolerance [8]. However, whether chicken CD4^+^CD25^+^ cells play a role in this progress is not clear. So we carried out some experiments to study the mechanism. In this experiment, we used less BSA (0.75 mg/egg) to induce immune tolerance than in the previous study (1.0 mg/egg). After hatching, chicks experienced four rounds of immunization and serum antibodies were analyzed. Results showed that humoral tolerance was induced. More experiments are needed to determine the minimum amount of exogenous protein that can induce immune tolerance.

Regulatory T cells (Tregs) play an important immunoregulation role to protect the host from excessive immune responses and maintain tolerance [23]. CD4^+^CD25^+^FoxP3^+^ Tregs is one population originated as a separate lineage of cells in the thymus [24]. Colleagues have found that there have immunoregulatory cells in the chicken thymus that function in B and T cell responses [25]. As the FoxP3 ortholog has yet to be identified in chickens, studying chicken CD4^+^CD25^+^ cells can help us to look inside avian Tregs. We produced an anti-chicken CD25 mAb as described in previous study [18]. In this study, we first identified the characteristics of chicken CD4^+^CD25^+^ cells. Results showed that they have no *IL-2* expression and high *IL-10* and *TGF-β* expression in CD4^+^CD25^+^ cells, and suppression characteristic in vitro. This finding is consistent with results of Shanmugasundaram who revealed that chicken CD4^+^CD25^+^ cells have similar characteristics to those in mammals [11]. In mammals, CD4^+^CD25^+^ Tregs can be derived from two sources, naturally occurring CD4^+^CD25^+^ cells which develop within the thymus and those generated in the periphery [26]. Furthermore, our previous study showed that peripheral blood lymphocytes could be activated by ConA to exert more CD4^+^CD25^+^ cells in vitro, suggesting that chicken CD4^+^CD25^+^ cells can be generated in the periphery [18]. We surmise that chicken CD4^+^CD25^+^ cells may be derived similarly to those in mammals, however more studies are needed to prove this hypothesis.

Interestingly, we found that during the immune tolerance induced by BSA injection, the percentage of CD4^+^CD25^+^ cells in peripheral blood lymphocytes are higher than the control group except Day 14 after hatch (Figure 3B). It is may be affected by the export wave of thymic CD4^+^CD25^+^ cells. CD4^+^CD25^+^ cells develop in the thymus and migrate to other organs such as the cecal tonsils and the spleen via blood circulation [15]. First export wave occurs at Day 1, and later export wave occurs after Day 7. We suggest that in the BSA injection group, the decrease of the CD4^+^CD25^+^ cell ratio in peripheral blood is not easy to bounce back after later export. However, more experimental evidence is needed to explore this conjecture.

Antibody depletion of cells in vivo has become a commonly employed method to determine the significance of a population of cells in a particular process, and more recently, to be a therapy to alleviate neoplastic and immune-mediated diseases [27]. In chickens, the traditional amniotic route of injecting anti-chicken CD25 antibodies costs more due to the amounts of antibodies required and the lack of direct contacts the embryos. Shanmugasundaram injected 0.5 mg anti-chicken CD25 mAb per egg into embryos at EID16 [16] or EID18 [17] to block the CD4^+^CD25^+^ cells. To the best of our knowledge, embryonic blood vessel injection of anti-chicken CD25 antibodies to reduce the CD4^+^CD25^+^ cells in chickens has not been reported in previous studies. The microinjection method by embryonic blood vessels in our study economized approximately 30-fold the amount of anti-chicken CD25 mAb and the deletion lasts longer than with the amniotic route. In our study, the injection of anti-chicken CD25 mAb started to reduce CD4^+^CD25^+^ cell percentage at 7 d post-hatch, that is 12 d post-injection. The decrease peaked at 21 d post-hatch, that is 26 d post-injection. The decrease lasted as long as 35 d post-hatch, that is 40 d post-injection. However, the results of Shanmugasundaram et al. [16] showed that the decrease of CD4^+^CD25^+^ cell percentage by amniotic injection of anti-chicken CD25 mAb started at 8 d post-injection, peaked at 12 d post-injection and disappeared at 20 d post-injection. As a whole, the decrease of CD4^+^CD25^+^ cell percentage caused by intravascular injection of anti-chicken CD25 mAb began later but last longer than that caused by amniotic injection. The blocking of CD4^+^CD25^+^ cells by anti-chicken CD25 mAb is not complete, maybe as there are proportional CD4^+^CD25^+^ cells in the spleen and cecal tonsils. Some pathogens may stimulate Tregs to escape host immune responses. In mammals, depletion of CD4^+^CD25^+^ cells by anti-CD25 antibody during bacterial and viral infections relieves the suppression of Treg and improves bacterial and viral clearance [28,29,30]. Anti-CD25 antibody-mediated Treg depletion has been applied to study the role of Tregs in several bacterial and viral infections in mammals. Our previous results showed that the Treg populations in the thymus and spleen decreased after IBDV infection, suggesting the role of Tregs in the pathogenesis of IBDV infections [18]. We can expect that anti-chicken CD25-induced Treg depletion is a probable way to improve anti-disease defense in chickens.

## 5. Conclusions

In our study, we innovatively discover that the intravascular injection of anti-chicken CD25 mAb on EID16 is effective to block chicken CD4^+^CD25^+^ cells. And to our knowledge, for the first time, chicken CD4^+^CD25^+^ cells are reported to play an essential role in maintaining the immunological tolerance in chickens inoculated with the xenogeneic antigen BSA on EID20.

## Figures and Tables

**Table 1:** Sequences of the primers used in qRT-PCR.

Gene	Direction	Sequence	GenBank Accession No.
GAPDH	Forward	GGTAGTGAAGGCTGCTGCTGAT	NM_204305.1
	Reverse	GGAGGAATGGCTGTCACCAT	
IL-2	Forward	TTCATCTCGAGCTCTACACACCAA	NM_204153.1
	Reverse	TGTCATCTTCAGTTTCTTTCTTCAGAGT	
IL-10 ^a^	Forward	GGCGACCTGGGCAACAT	NM_001004414.2
	Reverse	CCTTGATCTGCTTGATGGCTTT	
TGF-β ^b^	Forward	TGCGGCCAGATGAGCATATAG	M31154.1
	Reverse	GTGTCGGTGACATCGAAGGA	

^a,b^ Primers from Xiaoxue Yu [18].

**Table 2:** The relative expression of *IL-2*, *IL-10*, *TGF-β* mRNA in CD4^+^CD25^+^ cells.

Subgroups	*IL-2*	*IL-10*	*TGF-β*
CD4^+^CD25^−^ cells	1 ± 0.31	1 ± 0.23	1 ± 0.1
CD4^+^CD25^+^ cells	ND	41.18 ± 3.3	10.06 ± 1.7

The expression of *IL-2*, *IL-10*, *TGF-β* mRNA in CD4^+^CD25^+^ cells, gapdh as the reference gene. All mRNA contents were normalized to the mRNA content of the CD4^+^CD25^+^ group so that all bars represent fold increase or decrease compared with the CD4^+^CD25^−^ group, n = 3. Results are representative of two independent experiments (mean ± SEM). ND, not detectable.

## Supplementary Materials

The following are available online at https://www.mdpi.com/2306-7381/7/4/150/s1, A cartoon named “The simple protocol of microinjection” in mp4 format.
